# Supplementary material for: Single-Cell RNA Sequencing of the Testis of Ciona intestinalis Reveals the Dynamic Transcriptional Profile of Spermatogenesis in Protochordates
Source: Cells. 2022 Dec 8;11(24):3978. doi: 10.3390/cells11243978 (PMC9776925; doi:10.3390/cells11243978)
Supplement: Supplementary file 1 [file cells-11-03978-s001.zip › Supplementary Figures.pdf]

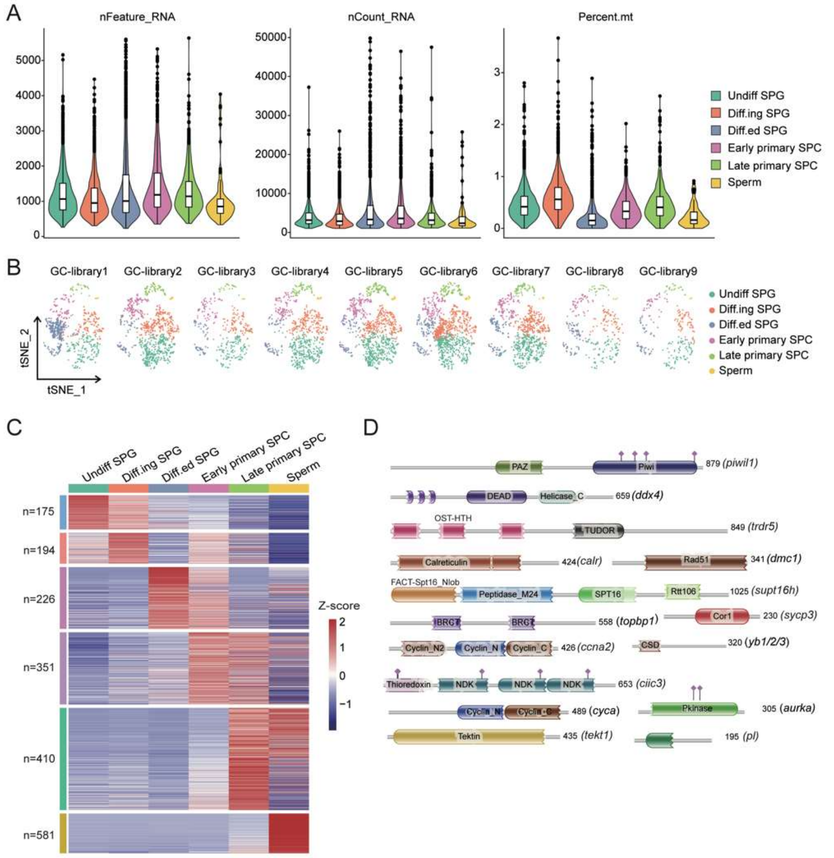


**Figure S1.** Basic summary about the single cell datasets (related to Figure1). (A) Violin plots for the distribution of the number of expressed genes, UMIs, and the percentage of mitochondrial UMIs. (B) Distribution of germ cells in each library. (C) Heatmap showing expression of all differentially expressed genes. (D) Display of structural domains of major marker genes.

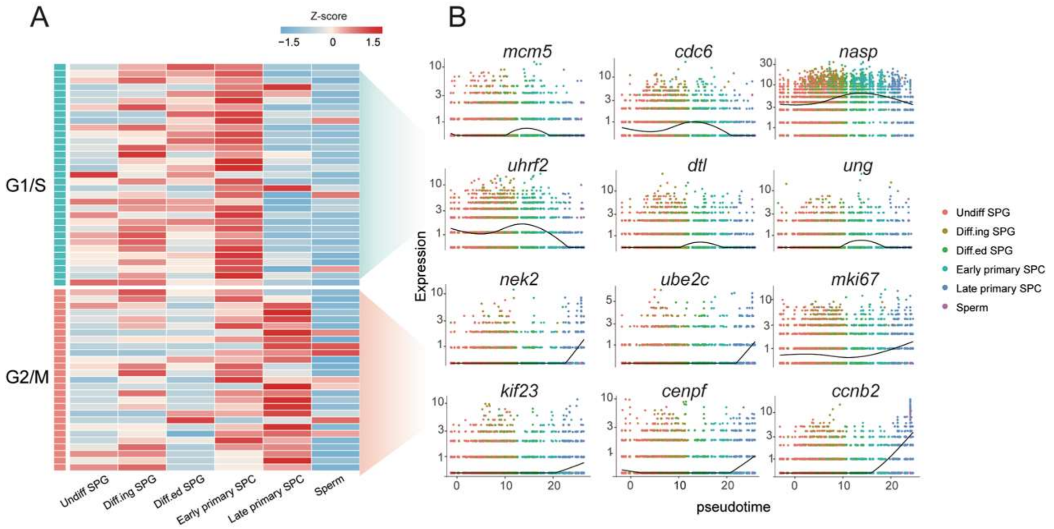


**Figure S2.** Expression analysis of cell cycle genes (related to Figure 2). (A) Heatmap showing expression of cell cycle genes. (B) The Expression Trend of Cell Cycle Genes.

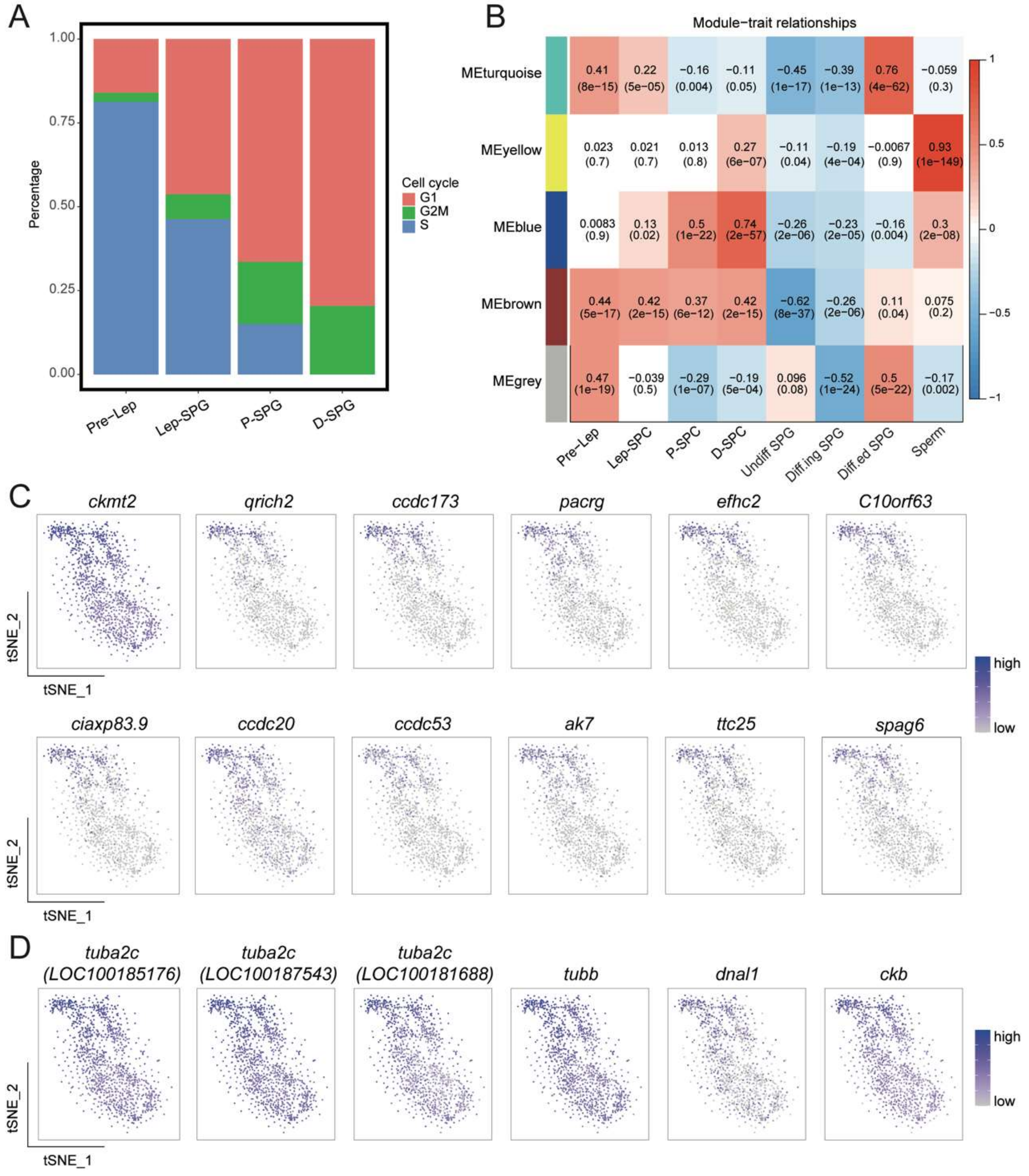


**Figure S3.** Gene expression analysis in modules (related to Figure 3). (A) Distribution of cell cycle status of spermatocyte. (B) Correlation between different gene modules and germ cell types. (C) Expression of blue module gene in spermatocytes. (D) Expression of brown module gene in spermatocytes.
